# Comparison of clinical outcomes between flexible antagonist protocol and long luteal phase protocol in patients with normal ovarian reserve function: a prospective cohort study

**DOI:** 10.3389/fendo.2025.1526895

**Published:** 2025-08-08

**Authors:** Ying Tian, Jing Hao, Xueliang Tu, Shaobin Feng, Mingtao Li, Yang Chen, Zelei Cao

**Affiliations:** Department of Reproductive Medicine, Yellow River Sanmenxia Affiliated Hospital of Henan University of Science and Technology, Sanmenxia, China

**Keywords:** ovarian stimulation protocols, clinical pregnancy, GnRH agonist, GnRH antagonist, IVF/ICSI outcomes, normal ovarian reserve

## Abstract

**Objective:**

Ovarian stimulation protocols play a pivotal role in the success of *in vitro* fertilization (IVF) and intracytoplasmic sperm injection (ICSI) treatments. This study compares the clinical outcomes of the long luteal phase GnRH agonist protocol and the flexible GnRH antagonist protocol in patients with normal ovarian reserve.

**Methods:**

This prospective cohort study was conducted at the Reproductive Medicine Center, Sanmenxia Hospital, Yellow River, from March 2021 to September 2023. Patients with normal ovarian reserve were enrolled and randomly assigned by a 1:3 ratio to either the long luteal phase protocol (Group A, n=42) or the flexible antagonist protocol (Group B, n=118). Data on patient characteristics, ovarian response, and embryological outcomes were collected and analyzed. Clinical outcomes, including clinical pregnancy, live birth rates, and ovarian hyperstimulation syndrome (OHSS) incidence, were assessed. Multivariate logistic regression was conducted to identify risk factors associated with clinical pregnancy.

**Results:**

There were no significant differences in baseline characteristics between the two groups (*P*>0.05). In terms of primary clinical outcomes, there were no significant differences in clinical pregnancy rate (54.8% *vs*. 56.8%, *P*=0.092), live birth rate (47.6% *vs*. 52.5%, *P*=0.278), or incidence of OHSS (0% *vs*. 2.5%, *P*=0.055) between Group A and Group B. Multivariable logistic regression analysis identified significant predictors of clinical pregnancy, including younger age (OR = 0.956, *P* = 0.042), higher AFC (OR = 1.127, *P* = 0.018), higher AMH levels (OR = 1.357, *P* = 0.005), greater endometrial thickness (OR = 1.162, *P* = 0.021), higher number of oocytes retrieved (OR = 1.234, *P* = 0.023), and better embryo quality (Grade I-II) (OR = 1.485, *P* = 0.002). No significant differences were observed between age-related subgroups (*P*>0.05), but success rates decreased with increasing age, highlighting age as a key factor influencing IVF/ICSI outcomes.

**Conclusion:**

The study found no significant differences in primary clinical outcomes between the two groups. However, younger age, higher AFC, higher AMH levels, greater endometrial thickness, higher number of oocytes retrieved, and better embryo quality were significant predictors of clinical pregnancy.

## Introduction

Infertility is a common and distressing condition affecting approximately 10-15% of reproductive-aged couples worldwide. Assisted reproductive technologies (ART), particularly *in vitro* fertilization (IVF) and intracytoplasmic sperm injection (ICSI), have revolutionized the management of infertility. Central to the success of IVF/ICSI is controlled ovarian hyperstimulation (COH), which aims to recruit multiple follicles to maximize the number of mature oocytes for fertilization (1). Optimal ovarian stimulation protocols are particularly crucial for achieving successful pregnancy outcomes, especially in patients with normal ovarian reserve, who typically have a favorable response to stimulation protocols (1, 2). However, the selection of the most appropriate stimulation protocol for this patient population remains a subject of ongoing debate.

The long luteal phase gonadotropin-releasing hormone agonist (GnRH-a) protocol has long been regarded as the standard approach for ovarian stimulation. This protocol involves the administration of a GnRH-a during the luteal phase of the preceding cycle to downregulate the hypothalamic-pituitary-ovarian axis, suppressing premature luteinizing hormone (LH) surges and allowing for more controlled follicular development. Ovarian stimulation is initiated after sufficient downregulation has been achieved, usually after 14 days of GnRH-a administration (3). Although this protocol has been associated with favorable clinical outcomes, including higher pregnancy and live birth rates, its extended duration and the potential for side effects such as ovarian hyperstimulation syndrome (OHSS) and flare-ups during downregulation pose challenges for some patients (4, 5). In contrast, the flexible GnRH antagonist protocol, developed as an alternative to the traditional GnRH-a protocol, offers a shorter and more patient-friendly approach. This protocol involves the administration of a GnRH antagonist during the follicular phase when the leading follicle reaches a diameter of 12–14 mm, effectively suppressing premature LH surges without the need for extended downregulation. The shorter duration of treatment and reduced risk of OHSS make the antagonist protocol an attractive option for both patients and clinicians (6). Additionally, the antagonist protocol has demonstrated comparable pregnancy outcomes to the agonist protocol in several studies (7, 8). However, concerns remain regarding its effectiveness in specific patient populations, such as those with normal ovarian reserve, where a more aggressive stimulation approach may be beneficial.

Patients with normal ovarian reserve, characterized by adequate antral follicle counts (AFC) and anti-Müllerian hormone (AMH) levels, typically respond well to ovarian stimulation. However, the optimal stimulation protocol for this population remains contentious. While the long luteal phase protocol may offer advantages in terms of controlled follicular development and a more predictable response, it is associated with higher risks of OHSS due to the recruitment of a larger number of follicles. The flexible antagonist protocol, on the other hand, may reduce the risk of OHSS but could lead to suboptimal ovarian response and lower pregnancy rates in some patients (9). As such, there is a need for robust comparative studies to determine which protocol is more effective in achieving successful pregnancy outcomes while minimizing adverse effects in patients with normal ovarian reserve. Recent studies have begun to shed light on this issue (10–13). This study aims to address these gaps by conducting a comprehensive comparison of clinical outcomes between the flexible antagonist protocol and the long luteal phase protocol in patients with normal ovarian reserve. Specifically, we seek to evaluate clinical pregnancy rates, live birth rates, and the incidence of OHSS, as well as secondary outcomes such as embryo quality, fertilization rates, and the number of oocytes retrieved.

## Patients and methods

### Study design

This prospective cohort study was conducted between March 2021 and September 2023 at the Reproductive Medicine Center, Sanmenxia Hospital, Yellow River. The study was complied with the guidelines of the Declaration of Helsinki and relevant national regulations. The study protocol has been reviewed and approved by the hospital’s institutional ethics committee (No: 2024LLS20240914043), and all participants have provided written informed consent.

### Population

**Figure 1:**
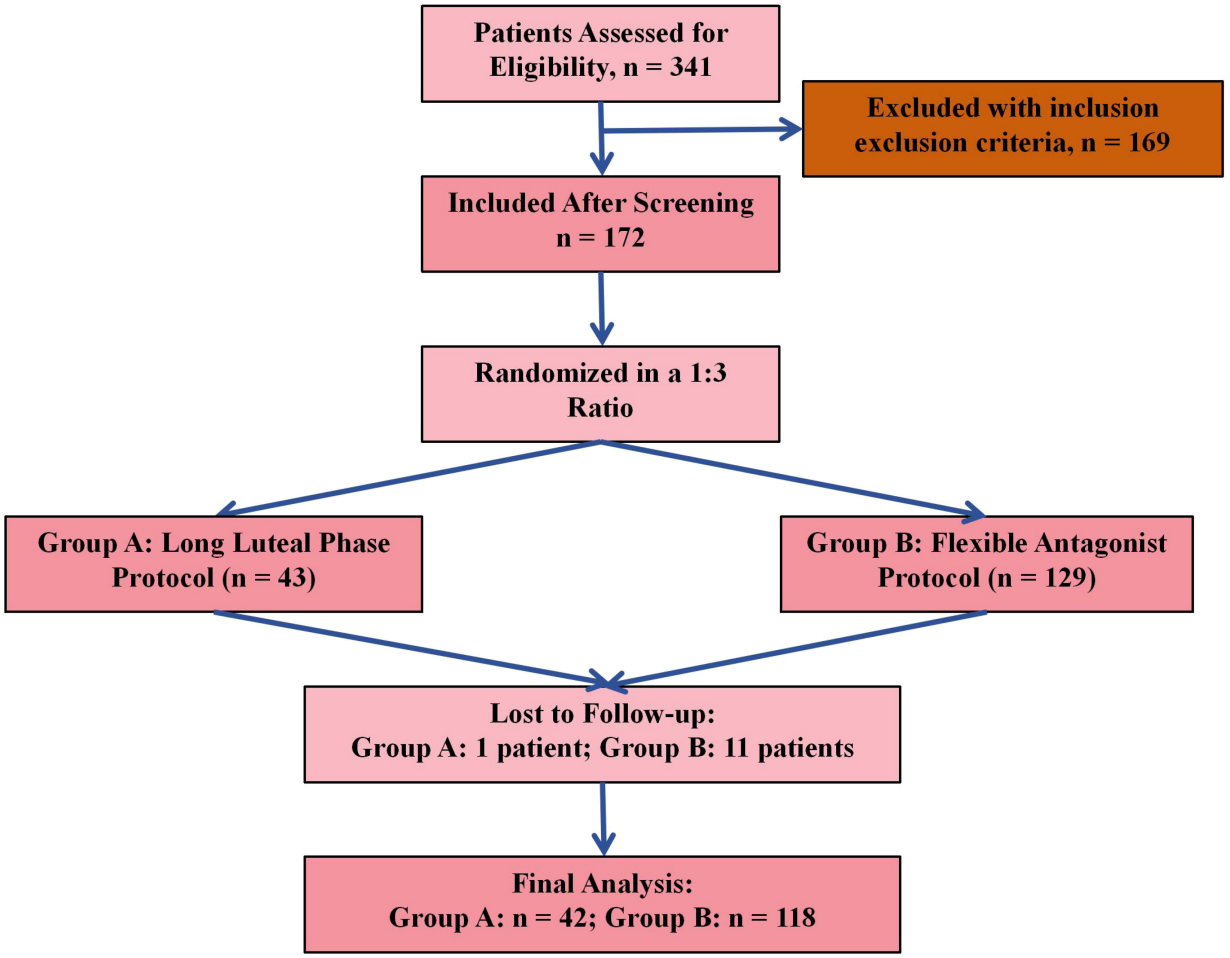
Flow chart.

A total of 341 patients were initially assessed for eligibility. Following the application of inclusion and exclusion criteria, 172 patients met the eligibility requirements and were included in the study. Participants were then allocated into two groups using a 1:3 ratio: 43 patients were assigned to Group A (Long Luteal Phase Protocol) and 129 to Group B (Flexible Antagonist Protocol). During follow-up, 1 patient in Group A and 11 patients in Group B were lost to follow-up. As a result, 42 patients in Group A and 118 patients in Group B were included in the final analysis (**Figure 1**). The 1:3 allocation ratio was based on the proportional use of protocols in clinical practice at our center during the study period. The flexible antagonist protocol (Group B) was more commonly utilized due to its shorter duration and lower risk of OHSS, whereas the long agonist protocol (Group A) was reserved for specific cases.

Inclusion Criteria were as follow: (1)Women included in this study were aged less than 40 years, with normal menstrual cycles (25–35 days) in the preceding three months, and AFC ranging from 8 to 15. (2)Baseline serum (FSH levels were ≤10 U/L, and AMH levels were >1.1 ng/mL. (3)All patients met the established criteria for IVF/ICSI treatment. Exclusion Criteria were as follow: (1) presence of chromosomal abnormalities in either partner, uterine abnormalities (e.g., unicornuate uterus, bicornuate uterus, or septate uterus), (2) any contraindications for IVF-embryo transfer (IVF-ET).

### Data collection

Data collection was meticulously carried out to record both biochemical and clinical outcomes following embryo transfer, including key demographic, clinical, and outcome variables. Baseline variables included patient age, body mass index (BMI), duration of infertility, AFC, AMH levels, and baseline serum FSH. During ovarian stimulation, data on gonadotropin dose, stimulation duration, endometrial thickness, estradiol and progesterone levels on trigger day, and the number of follicles ≥18 mm were recorded. Embryology-related variables included the number of oocytes retrieved, mature oocyte count, fertilization rate, and embryo quality on day 3.

### Treatment protocols

Long Luteal Phase Protocol: this protocol involved the initiation of a GnRH agonist in the mid-luteal phase of the preceding menstrual cycle to achieve downregulation of the hypothalamic-pituitary-ovarian axis. After 14 days of downregulation, COH was commenced using recombinant human FSH (rFSH) or urinary-derived gonadotropins. The gonadotropin dose was adjusted based on patient age, body weight, and AFC. Ovulation was triggered with human chorionic gonadotropin (hCG) once at least three follicles reached a diameter of ≥18 mm, and oocytes were retrieved 36 hours after the trigger.

Flexible Antagonist Protocol: this protocol began with COH initiated on day 2 or 3 of the menstrual cycle. The starting dose of gonadotropins was individualized according to ovarian response as assessed by transvaginal ultrasound (B-ultrasound) and serum estradiol (E2) levels. When the leading follicle reached 12 mm in diameter, a GnRH antagonist was administered to prevent premature LH surges. Ovulation was triggered using a dual trigger (hCG combined with a GnRH agonist) once two or more follicles reached ≥18 mm, and oocyte retrieval occurred 36 hours post-trigger.

### Embryo assessment and transfer

Embryos were assessed on day 3 post-fertilization according to standard morphologic criteria. Grading was based on the number of blastomeres, degree of fragmentation, and uniformity of blastomere size, with grades I and II considered high-quality embryos. Embryos of higher quality were prioritized for transfer. In cases of a high risk for OHSS— characterized by serum E2 levels exceeding 4000 pg/mL or retrieval of more than 15 oocytes— a freeze-all strategy was employed. This involved cryopreservation of all viable embryos and postponement of the embryo transfer to a subsequent frozen embryo transfer (FET) cycle to minimize the risk of OHSS.

### Study outcome

Patients with confirmed clinical pregnancies were followed at regular intervals to ensure continued pregnancy viability and to monitor for complications. The first follow-up was conducted at 12 weeks of gestation, focusing on the viability of the pregnancy and the assessment of early fetal development. A second follow-up was carried out at 24 weeks of gestation to further assess fetal growth and maternal health. Finally, all patients were followed up 2 weeks postpartum to document delivery outcomes, including birth weight, gestational age at delivery, and any neonatal complications.

The primary outcomes included: (1) Clinical pregnancy rate: defined as the number of clinical pregnancy cycles per number of transfer cycles × 100%. Clinical pregnancy was confirmed by the presence of at least one gestational sac with a fetal heartbeat detected via transvaginal ultrasound at 6–8 weeks of gestation. This calculation includes both fresh embryo transfer cycles and the first frozen embryo transfer (FET) cycle for patients undergoing a freeze-all strategy, with each patient contributing one transfer cycle. (2) Live birth rate: defined as the number of live births per number of transfer cycles × 100%. A live birth is defined as the delivery of at least one living infant at or beyond 24 weeks of gestation. This calculation includes both fresh embryo transfer cycles and the first FET cycle for patients undergoing a freeze-all strategy, with each patient contributing one transfer cycle, representing a single-cycle liver birth rate. (3) Incidence of moderate to severe OHSS: classified based on standard criteria including symptom severity, ovarian enlargement, and biochemical markers.

The secondary outcomes included: (1) Oocyte metrics: total number of oocytes retrieved, mature oocyte rate (number of mature oocytes per total oocytes retrieved), and fertilization rate. (2) Blastocyst formation rate: The percentage of embryos progressing to the blastocyst stage. (3) Embryo quality: proportion of high-quality embryos (grades I and II). (4) Implantation rate: the number of gestational sacs per number of embryos transferred × 100%. (5) Miscarriage rates: early miscarriage rate (spontaneous loss of pregnancy within 12 weeks) and overall miscarriage rate (spontaneous loss of pregnancy at any gestational age).

### Sample size calculation

The sample size was calculated by Pass 11.0 and aimed to detect a 15% difference in clinical pregnancy rate (50% for the long luteal phase protocol *vs*. 35% for the flexible antagonist protocol) based on prior studies (14, 15). Using a two-proportion z-test with 80% power, a two-sided α = 0.05, and a 1:3 allocation ratio, 144 patients (36 in Group A and 108 in Group B) were required. Accounting for a 10% dropout rate, the target enrollment was increased to 160 patients (40 in Group A and 120 in Group B).

### Statistical analysis

Continuous variables were described as mean ± standard deviation (SD) for normally distributed data. For data that were not normally distributed, variables were expressed as the median and interquartile range (IQR). Comparisons between the two groups were conducted using independent t-tests for normally distributed variables and the Mann-Whitney U test for non-normally distributed variables. Categorical variables were expressed as percentages or proportions. Comparisons between groups were made using chi-square tests or Fisher’s exact test.

Multivariable logistic regression analysis was conducted to identify independent predictors of clinical pregnancy, adjusting for potential confounders. Variables included in the model (age, BMI, AFC, AMH, gonadotropin dose, number of oocytes retrieved, and embryo quality [Grade I-II]) were selected *a priori* based on their established clinical relevance to IVF/ICSI outcomes, and their potential associations in univariate analyses (*P* < 0.10). The grouping factor (long luteal phase *vs*. flexible antagonist protocol) was excluded from the model because the primary study objective was to compare protocol outcomes directly, and the exploratory analyses confirmed that the protocol type was not a significant predictor (*P* > 0.05). Adjusted odds ratios (ORs) with 95% confidence intervals (CIs) were reported.

A subgroup analysis by age (>20–25, 30–35, and 35–<40 years) was performed to assess clinical pregnancy and live birth rates within each age stratum, comparing Group A and Group B using chi-square tests or Fisher’s exact test. All statistical analyses were performed using the latest version of SPSS (IBM, Chicago, USA). A two-tailed *P* value of less than 0.05 was considered statistically significant.

## Results

### Comparison of basic characteristics

**Table 1** showed no significant differences in baseline characteristics between Group A and Group B. Age, BMI, duration of infertility, AFC, AMH, FSH levels, gonadotropin dose, stimulation duration, number of follicles ≥18 mm, oocytes retrieved, mature oocyte count, fertilization rates, and day 3 embryo quality were comparable across both groups (*P*>0.05).

**Table 1:** Comparison of basic characteristics between the two groups.

Variables	Group A (n=42)	Group B (n=118)	P value
Age (years)	31.2 ± 3.5	30.9 ± 3.6	0.641
BMI (kg/m²)	24.6 ± 2.3	24.8 ± 2.2	0.618
Duration of infertility (years)	3.8 ± 1.7	3.6 ± 1.6	0.495
AFC	10.8 ± 2.1	11.0 ± 2.2	0.609
AMH (ng/mL)	3.4 ± 0.7	3.5 ± 0.8	0.474
FSH (U/L)	7.5 ± 1.2	7.6 ± 1.3	0.663
Gonadotropin dose (IU)	2108.3 ± 350.4	2028.7 ± 316.2	0.175
Stimulation duration (days)	10.2 ± 1.3	9.8 ± 1.5	0.127
Estradiol on trigger day (pg/mL)	3021.6 ± 508.7	2954.7 ± 460.2	0.433
Endometrial thickness (mm)	10.5 ± 1.8	10.7 ± 1.7	0.723
Progesterone on trigger day (ng/mL)	1.0 ± 0.3	1.1 ± 0.3	0.689
Number of follicles ≥18 mm	9.4 ± 2.1	9.6 ± 2.0	0.584
Number of oocytes retrieved	11.8 ± 2.7	12.0 ± 2.5	0.664
Mature oocyte count	10.2 ± 2.3	10.4 ± 2.1	0.606
Fertilization rate (%)	75.6 ± 10.1	74.2 ± 11.5	0.485
Day 3 embryo quality (Grade I-II) (%)	30 (71.4%)	87 (73.7%)	0.774

### Comparison of clinical outcomes

**Figure 2:**
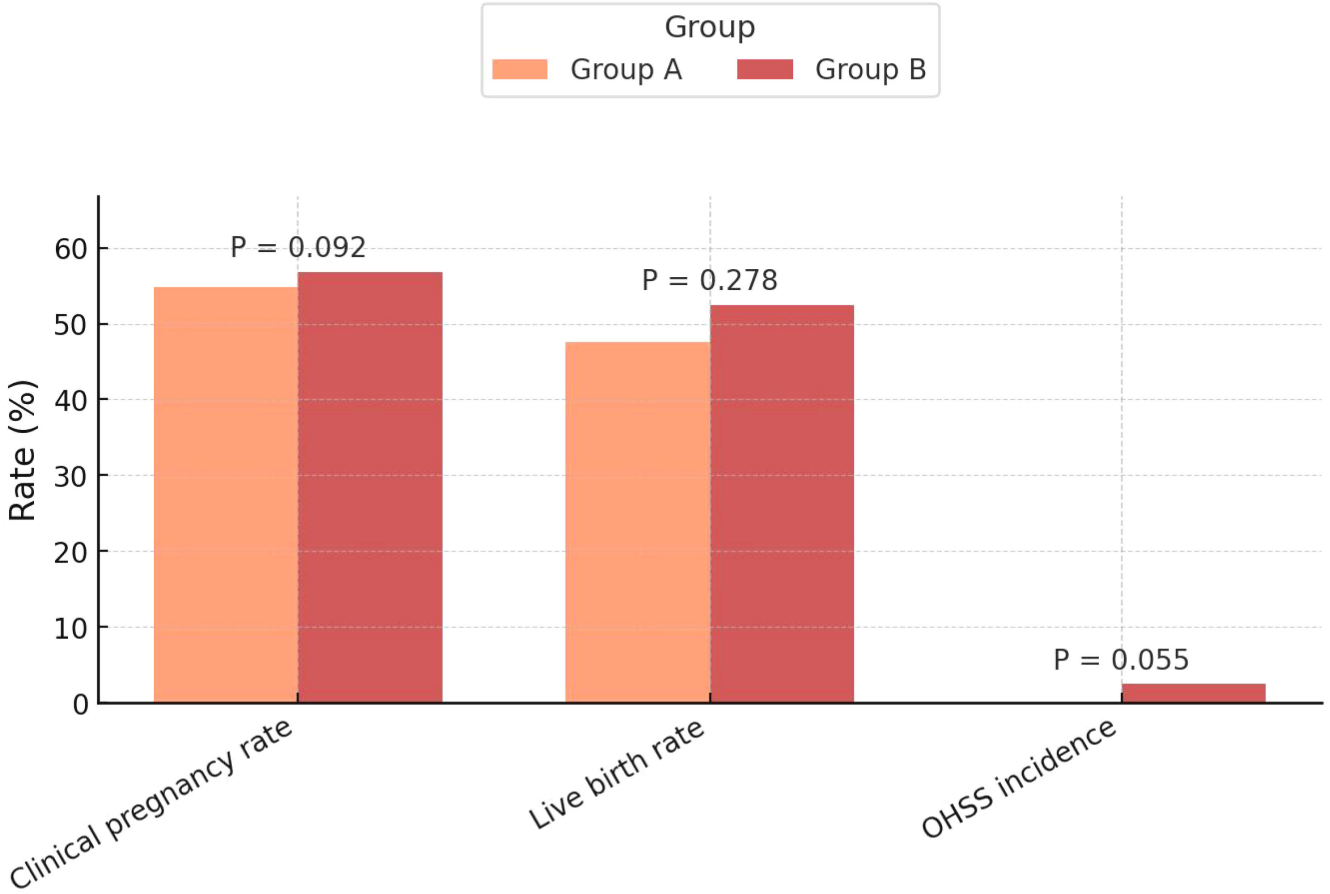
Comparison of primary clinical outcomes between the two groups.

The clinical pregnancy rate was similar between groups (54.8% *vs*. 56.8%, *P*=0.092), with fresh cycle rates of 53.3% *vs*. 56.5% and FET cycle rates of 58.3% *vs*. 57.6%. Likewise, the live birth rate showed no significant difference (47.6% *vs*. 52.5%, *P*=0.278), with fresh cycle rates of 46.7% *vs*. 52.9% and FET cycle rates of 50.0% *vs*. 51.5%. These findings indicate comparable efficacy of the two protocols in achieving clinical pregnancy and live births. Additionally, the incidence of OHSS was low, with no cases in Group A and 2.5% in Group B, but this difference did not reach statistical significance (*P*=0.055) (**Table 2**, **Figure 2**).

**Table 2:** Comparison of clinical outcomes between the two groups.

Clinical outcome	Group A (n=42)	Group B (n=118)	P value
Primary outcome			
Clinical pregnancy rate (%)	23 (54.8%)	67(56.8%)	0.092
Fresh cycles (n)	16/30 (53.3%)	48/85 (56.5%)	
FET cycles (n)	7/12 (58.3%)	19/33 (57.6%)	
Live birth rate (%)	20 (47.6%)	62 (52.5%)	0.278
Fresh cycles (n)	14/30 (46.7%)	45/85 (52.9%)	
FET cycles (n)	6/12 (50.0%)	17/33 (51.5%)	
OHSS incidence (%)	0	3 (2.5%)	0.055
Secondary outcome	Group A (n=42)	Group B (n=118)	
Embryos transferred	1.8 ± 0.4	1.9 ± 0.3	0.093
Implantation rate (%)	19 (45.2%)	50 (42.4%)	0.447
Early miscarriage rate (%)	2 (4.8%)	9(7.6%)	0.329
Overall miscarriage rate (%)	3 (7.1%)	15 (12.7%)	0.127
Blastocysts formed	5.2 ± 2.0	5.4 ± 1.8	0.549
Day 5 blastocyst formation rate (%)	16 (38.1%)	47 (39.8%)	0.643

**Figure 3:**
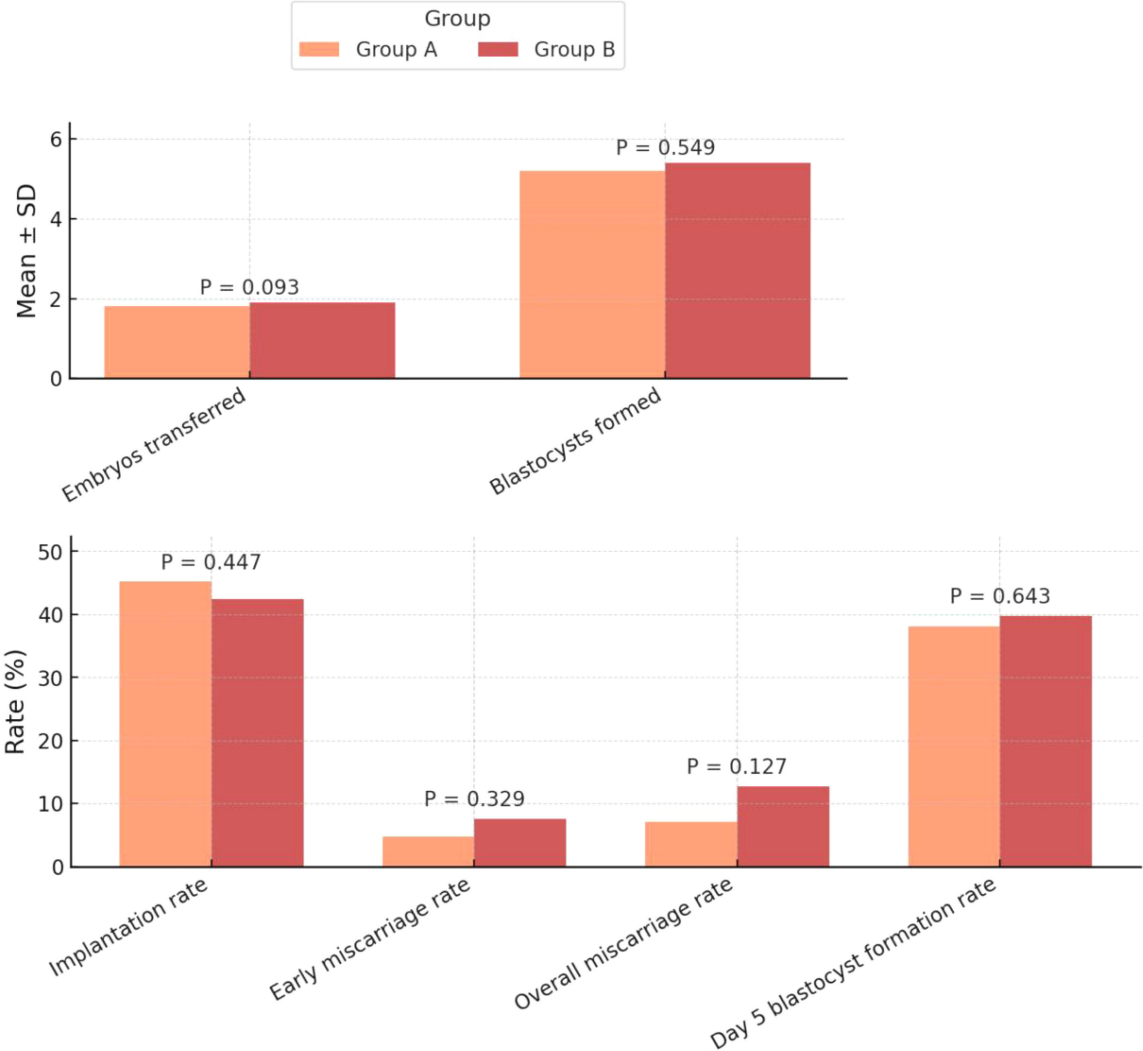
Comparison of secondary clinical outcomes between the two groups.

For the secondary outcomes, there were no significant differences in the number of embryos transferred (1.8 ± 0.4 *vs*. 1.9 ± 0.3, *P*=0.093) (**Figure 3**), implantation rate (45.2% *vs*. 42.4%, *P*=0.447), or early miscarriage rate (4.8% *vs*. 7.6%, *P*=0.329) (**Figure 3**). Similarly, the overall miscarriage rate (7.1% *vs*. 12.7%, *P*=0.127), the number of blastocysts formed (5.2 ± 2.0 *vs*. 5.4 ± 1.8, *P*=0.549), and the day 5 blastocyst formation rate (38.1% *vs*. 39.8%, *P*=0.643) were all comparable between the two groups (**Table 2**).

### Logistic regression analysis of risk factors for clinical pregnancy

The univariate logistic regression analysis (**Table 3**) revealed several significant predictors of clinical pregnancy (*P* < 0.05). Younger age (*OR* = 0.940, *P* = 0.035), lower BMI (OR = 0.950, *P* = 0.045), higher AFC (OR = 1.150, P = 0.015), higher AMH levels (OR = 1.400, *P* = 0.008), greater endometrial thickness (OR = 1.180, *P* = 0.018), higher gonadotropin dose (OR = 1.003, *P* = 0.044), higher estradiol on trigger day (OR = 1.002, *P* = 0.050), lower progesterone on trigger day (OR = 0.820, *P* = 0.031), greater number of oocytes retrieved (OR = 1.250, *P* = 0.020), higher mature oocyte count (OR = 1.220, *P* = 0.025), and better embryo quality (Grade I-II) (OR = 1.600, *P* = 0.010) were significantly associated with increased odds of clinical pregnancy. Additionally, duration of infertility (OR = 0.900, *P* = 0.090), FSH levels (OR = 0.920, *P* = 0.080), and fertilization rate (OR = 1.030, *P* = 0.070) showed marginal associations (*P* < 0.10).

**Table 3:** Univariable logistic regression for selected predictors of clinical pregnancy.

Variables	β	SE	OR	95% CI	P value
Age (years)	-0.062	0.029	0.940	0.888 - 0.995	0.035
BMI (kg/m²)	-0.051	0.025	0.950	0.904 - 0.998	0.045
Duration of infertility (years)	-0.105	0.062	0.900	0.797 - 1.017	0.090
AFC	0.140	0.057	1.150	1.029 - 1.286	0.015
AMH (ng/mL)	0.336	0.126	1.400	1.094 - 1.792	0.008
FSH (U/L)	-0.083	0.047	0.920	0.839 - 1.009	0.080
Gonadotropin Dose (IU)	0.003	0.002	1.003	1.001 - 1.008	0.044
Estradiol on Trigger Day (pg/mL)	0.002	0.001	1.002	1.000 - 1.004	0.050
Endometrial Thickness (mm)	0.166	0.070	1.180	1.029 - 1.354	0.018
Progesterone on Trigger Day (ng/mL)	-0.198	0.091	0.820	0686 - 0.980	0.031
Number of Oocytes Retrieved	0.223	0.096	1.250	1.035 - 1.509	0.020
Mature Oocyte Count	0.199	0.088	1.220	1.026 - 1.451	0.025
Fertilization Rate (%)	0.029	0.016	1.030	0.998 - 1.063	0.070
Embryo Quality (Grade I-II)	0.470	0.182	1.600	1.120 - 2.286	0.010

Variables with *P* < 0.10 in the univariate logistic regression analysis were included in the multivariable logistic regression analysis, and the results (**Table 4**) demonstrated that younger age (OR = 0.956, *P* = 0.042), higher AFC (OR = 1.127, *P* = 0.018), higher AMH levels (OR = 1.357, *P* = 0.005), greater endometrial thickness (OR = 1.162, *P* = 0.021), greater number of oocytes retrieved (OR = 1.234, *P* = 0.023), and better embryo quality (Grade I-II) (OR = 1.485, *P* = 0.002) were significant independent predictors of clinical pregnancy (*P* < 0.05). Gonadotropin dose (OR = 1.002, *P* = 0.050) showed trends toward significance but did not reach statistical significance (*P* > 0.05).

**Table 4:** Multivariable logistic regression for selected predictors of clinical pregnancy.

Variables	β	SE	OR	95% CI	P value
Age (years)	-0.045	0.020	0.956	0.920 - 0.993	0.042
AFC	0.120	0.045	1.127	1.035 - 1.227	0.018
AMH (ng/mL)	0.305	0.090	1.357	1.137 - 1.620	0.005
Gonadotropin Dose (IU)	0.002	0.001	1.002	1.000 - 1.004	0.050
Endometrial Thickness (mm)	0.150	0.065	1.162	1.023 - 1.320	0.021
Number of Oocytes Retrieved	0.210	0.065	1.234	1.084 - 1.408	0.023
Embryo Quality (Grade I-II)	0.395	0.075	1.485	1.265 - 1.742	0.002

### Subgroup analyses

**Table 5** presents the age-related subgroup analysis of clinical pregnancy and live birth rates for Group A and Group B, stratified into >20–25, 30–35, and 35–<40 years subgroups. In the >20–25 years subgroup, clinical pregnancy rates were 66.7% (Group A, n=6) *vs*. 72.2% (Group B, n=18, *P*=0.706), and live birth rates were both 66.7% (*P*=1.000). For the 30–35 years subgroup, clinical pregnancy rates were 57.1% (Group A, n=28) *vs*. 57.7% (Group B, n=78, *P*=0.953), and live birth rates were 50.0% *vs*. 53.8% (*P*=0.717). In the 35–<40 years subgroup, clinical pregnancy rates were 37.5% (Group A, n=8) *vs*. 40.9% (Group B, n=22, P=0.861), and live birth rates were 25.0% *vs*. 36.4% (*P*=0.684). No significant differences were observed between groups, but success rates decreased with increasing age, highlighting age as a key factor influencing IVF/ICSI outcomes.

**Table 5:** Age-Related subgroup analysis of clinical pregnancy and live birth rates.

Age Subgroup	Outcome	Group A	Group B	P value
>20–25 years		(n=6)	(n=18)	
	Clinical pregnancy rate (%)	4/6 (66.7%)	13/18 (72.2%)	0.706
	Live birth rate (%)	4/6 (66.7%)	12/18 (66.7%)	1.000
30–35 years		(n=28)	(n=78)	
	Clinical pregnancy rate (%)	16/28 (57.1%)	45/78 (57.7%)	0.953
	Live birth rate (%)	14/28 (50.0%)	42/78 (53.8%)	0.717
35–<40 years		(n=8)	(n=22)	
	Clinical pregnancy rate (%)	3/8 (37.5%)	9/22 (40.9%)	0.861
	Live birth rate (%)	2/8 (25.0%)	8/22 (36.4%)	0.684

Clinical pregnancy rate and live birth rate are calculated as the number of events divided by the number of embryo transfer cycles within each age subgroup (one per patient, including both fresh and first frozen embryo transfer [FET] cycles).

## Discussion

This study compares two commonly used ovarian stimulation protocols— the long luteal phase GnRH agonist protocol and the flexible GnRH antagonist protocol— in patients with normal ovarian reserve undergoing IVF/ICSI. Our findings demonstrate no significant differences between the two protocols in terms of clinical pregnancy rates, live birth rates, or incidence of OHSS. Significant predictors of clinical pregnancy were identified, including AFC, AMH levels, endometrial thickness, number of oocytes retrieved, and embryo quality, while increasing age negatively impacted pregnancy outcomes.

Our study is consistent with the growing body of literature demonstrating that both the GnRH agonist and antagonist protocols are effective for ovarian stimulation in patients with normal ovarian reserve (14–16). However, the flexibility and patient-centered advantages of the antagonist protocol, such as shorter treatment duration and lower risk of OHSS, suggest that it may be preferable for many patients (17–19). Our findings align with several studies that have found no significant differences in pregnancy or live birth rates between the GnRH agonist and antagonist protocols in patients with normal ovarian reserve (20–23). Lambalk et al. (14) and Venetis et al (15) both conducted systematic reviews and meta-analyses, reporting that although antagonist protocols lead to shorter treatment cycles and lower OHSS rates, pregnancy outcomes are comparable between the protocols. Similarly, Kadoura et al. (24) found no differences in live birth rates between the two protocols, though the antagonist protocol reduced patient discomfort and treatment burden. The absence of significant differences in OHSS incidence in our study is supported by earlier findings from studies such as those by Olivennes et al. (25), which suggested that the antagonist protocol is particularly beneficial in preventing severe OHSS in high responders. A systematic review by Engmann et al. (26) reinforced this view, demonstrating the antagonist protocol’s role in enhancing safety without compromising efficacy.

Our findings of equivalent clinical outcomes between the GnRH agonist and antagonist protocols in patients with normal ovarian reserve resolve apparent contradictions in the literature. While some studies suggest advantages for the agonist protocol in populations with diminished ovarian reserve or recurrent implantation failure (27, 28), others report comparable efficacy across protocols in broader populations (14, 15). The equivalence observed in our study is attributable to the focus on patients with normal ovarian reserve, who respond well to both protocols, as evidenced by similar clinical pregnancy (54.8% *vs*. 56.8%, *P*=0.092) and live birth rates (47.6% *vs*. 52.5%, *P*=0.278). Systematic reviews by Lambalk et al. (14) and Venetis et al. (15) support this equivalence, demonstrating no significant differences in live birth rates, while Kadoura et al. (24) further confirm comparable outcomes in polycystic ovary syndrome patients, a group with robust ovarian response. The antagonist protocol’s shorter treatment duration and lower OHSS incidence, as noted by Nie et al. (20), enhance its appeal without compromising efficacy, providing clinicians with flexibility to tailor treatments based on patient preferences and risk profiles.

The absence of protocol-specific differences in our study contrasts with studies reporting advantages for one protocol over the other, a discrepancy attributable to differences in patient populations and study methodologies (14, 15, 27, 28). Studies observed improved outcomes with the agonist protocol in patients with diminished ovarian reserve, where prolonged downregulation may enhance follicular synchronization (27, 28). In contrast, our study’s focus on patients with normal ovarian reserve, who exhibit robust responses to both protocols, likely minimizes differential effects. Additionally, our prospective design with randomization and standardized clinical protocols, such as endometrial thickness ≥8 mm and a freeze-all strategy for high-risk cases, reduced variability that may amplify protocol-specific differences in retrospective or heterogeneous studies. Systematic reviews (14, 15) corroborate that protocol equivalence is more pronounced in normal responders, supporting our findings and highlighting the importance of patient selection in ART outcomes.

The results suggest that both protocols are equally effective for patients with normal ovarian reserve, providing flexibility in treatment options. The flexible antagonist protocol offers distinct advantages, including reduced treatment burden and a lower incidence of severe OHSS. These benefits are particularly relevant in modern clinical practice, where patient comfort and safety are paramount. The role of AFC, AMH, and embryo quality as predictors of success in IVF/ICSI cycles has been well documented in previous studies. AFC and AMH have been shown to be reliable markers of ovarian reserve and predictors of ovarian response to stimulation (29). Our findings further emphasize the importance of these markers in guiding treatment decisions, particularly in identifying patients who may benefit from individualized gonadotropin dosing. Age remains a critical factor influencing clinical pregnancy outcomes, as demonstrated by the negative association between increasing age and pregnancy success in our study. This is consistent with the extensive literature highlighting the impact of maternal age on oocyte quality and reproductive outcomes (30, 31). As age increases, the cumulative impact of genetic and cellular changes within oocytes leads to reduced implantation potential and increased miscarriage rates (32). Although ovarian reserve markers like AFC and AMH provide valuable information, they cannot entirely mitigate the age-related decline in fertility.

Additionally, endometrial factors, such as endometrial thickness, and hormonal levels, such as progesterone on trigger day, are critical for pregnancy success, particularly in fresh embryo transfer cycles (33). In our study, endometrial thickness was standardized (≥8 mm) for fresh transfers, and progesterone levels were monitored to defer fresh transfers in cases of elevation (>1.5 ng/mL), employing a freeze-all strategy to optimize outcomes. These factors were not included in the logistic regression model (**Table 3**) due to their standardization across groups and lack of significant differences in preliminary analyses. However, we acknowledge that variability in endometrial receptivity or subtle progesterone elevations may still influence outcomes, and their exclusion from the regression analysis is a limitation. Future studies should incorporate these factors as variables to further elucidate their impact on pregnancy outcomes in patients with normal ovarian reserve.

Our findings have several important implications for clinical practice. The comparable efficacy of the two protocols means that clinicians have the flexibility to select a protocol based on patient preferences, clinical workflow, and the risk of OHSS. The antagonist protocol, with its shorter duration and lower complication risk, may be more suitable for patients seeking a less invasive treatment approach (33). The importance of embryo quality in predicting clinical pregnancy further emphasizes the need for careful embryo selection and grading during IVF/ICSI cycles. As demonstrated in several studies, morphologic criteria remain a critical component of embryo assessment, contributing significantly to treatment success. Our study also highlights the value of tailoring ovarian stimulation protocols to individual patient characteristics, including age and ovarian reserve markers. By adjusting stimulation protocols based on these factors, clinicians can optimize outcomes while minimizing the risk of complications such as OHSS.

### Strengths and limitations

This study’s prospective design and large sample size are key strengths, allowing for rigorous data collection and robust comparisons between the two protocols. The random assignment of patients to each protocol minimized selection bias and enhanced the generalizability of the findings to other patient populations with normal ovarian reserve (34). The randomization methhod ensured balanced baseline characteristics and minimized the selection bias. Additionally, the use of multivariable logistic regression further adjusted for potential confounders, reducing the risk of bias in identifying predictors of clinical pregnancy.

However, there are several limitations to consider. First, the study was conducted at a single center, which may limit the external validity of the findings. Multi-center studies are needed to confirm these results in more diverse populations and clinical settings. Second, while the sample size was sufficient for the primary outcome, it may be considered moderate for detecting smaller differences in secondary outcomes, such as implantation or blastocyst formation rates, potentially limiting the study’s power for these endpoints. Nevertheless, the observed non-significant differences in primary outcomes (*P* > 0.05) suggest that the sample size was appropriate for the study’s main objectives. Additionally, while the study focused on short-term outcomes such as clinical pregnancy and live birth rates, it did not assess long-term neonatal outcomes or maternal health beyond the postpartum period. These outcomes are crucial for evaluating the overall safety and effectiveness of ovarian stimulation protocols. Another limitation is the potential for unmeasured confounders, such as genetic factors or variations in laboratory techniques, that may have influenced the outcomes. Future studies should aim to control for these variables and include more detailed assessments of patient and embryological characteristics.

Future research should focus on further refining ovarian stimulation protocols to improve patient outcomes and safety (35, 36). Randomized controlled trials with larger, more diverse populations are needed to confirm the equivalence of the GnRH agonist and antagonist protocols in different patient groups, including those with diminished ovarian reserve and those undergoing multiple IVF cycles.

## Conclusion

In conclusion, this prospective cohort study found no significant differences in clinical pregnancy or live birth rates between the long luteal phase GnRH agonist protocol and the flexible GnRH antagonist protocol in patients with normal ovarian reserve undergoing IVF/ICSI. While both protocols are effective, the flexible antagonist protocol offers a more patient-friendly approach, with shorter treatment duration and lower risk of OHSS. Clinicians should consider individual patient characteristics, including age, ovarian reserve markers, endometrial factors, and personal preferences, when selecting the most appropriate protocol. Further research is needed to confirm these findings in larger, multi-center studies and to explore the long-term outcomes associated with different ovarian stimulation protocols.

## Data Availability

The raw data supporting the conclusions of this article will be made available by the authors, without undue reservation.
